# Age and seasons influence on at-home pulse oximetry results in children evaluated for suspected obstructive sleep apnea

**DOI:** 10.1186/s13052-017-0428-y

**Published:** 2017-12-04

**Authors:** Martino Pavone, Elisabetta Verrillo, Nicola Ullmann, Serena Caggiano, Valentina Negro, Renato Cutrera

**Affiliations:** Pediatric Pulmonology & Respiratory Intermediate Care Unit, Sleep and Long Term Ventilation Unit, Academic Department of Pediatrics (DPUO), Pediatric Hospital “Bambino Gesù” Research Institute, Piazza S. Onofrio 4, 00165 Rome, Italy

**Keywords:** Pulse oximetry, Obstructive sleep apnea, Seasonal variability

## Abstract

**Background:**

Seasonal variability on obstructive sleep apnea has already been studied by polysomnography in children. Winter and spring season emerged as critical periods. No data are currently available for pulse oximetry performed at home. The aim of our study was to evaluate the effect of seasonality and age on the results of at-home pulse oximetry performed in children referred for suspected OSA.

**Methods:**

We retrospectively studied 781 children (64.3% Males), aged 4.9 ± 2.5 years. For all patients, we evaluated both pulse oximetry metrics and the McGill Oximetry Score. Variables for seasonal groups were assessed using Kruskal-Wallis test. A logistic regression model was performed to assess the relationship between patients’ main characteristics, season period and the likelihood to have an abnormal McGill Oximetry Score.

**Results:**

Patients recorded during winter were significantly younger (*p* < 0.02), nadir SpO_2_ was significantly lower (*p* < 0.002) and DI_4_ significantly higher than during others seasons (*p* < 0.005). Moreover, patients recorded during winter were nearly 2 times more likely to have an abnormal MOS (aOR 1.949). The logistic regression showed that also younger age (*p* < 0.0001) was associated with a higher risk to find an abnormal pulse oximetry.

**Conclusions:**

In our study, the winter season confirms to be a critical period for pulse-oximetry and it should be taken into account by clinicians for a correct interpretation of tests. Our data show that also younger age affects the prevalence of abnormal at-home pulse oximetry in children.

## Background

Obstructive Sleep Apnea (OSA) in children is characterized by partial and/or intermittent complete upper airway obstruction that may disrupt normal sleep ventilation and EEG typical patterns [1].

The prevalence of OSA in children varies according with different studies from 1 to 5% [2–4].

Adenotonsillar hypertrophy is the most common risk factor for developing OSA in children. However, growing attention has been pointed out to other risk factors such as obesity, craniofacial anomalies, and neuromuscular disease [1–4].

Symptoms include snoring, laboured breathing, witnessed apnea, profuse sweating, disturbed sleep, and daytime neurobehavioral problems.

Untreated OSA may lead to severe complications as a consequence of intermittent hypoxia/re-oxygenation/hypercapnia, arousals and intra-thoracic pressure variation episodes [5]. Complications include neurocognitive impairment, behavioural, cardiovascular, and metabolic issues [5, 6].

Polysomnography (PSG) in a sleep laboratory represents the gold standard diagnostic exam for OSA. However, PSG is a costly and time-consuming procedure. Moreover, PSG may have difficult clinical application due to limited availability and long waiting lists [1, 2, 7].

To obviate these important issues, pulse oximetry is an increasingly used abbreviated testing modality for the evaluation of children with suspected OSA [8–12]. Brouillette et al. [9], demonstrated that an abnormal nocturnal pulse oximetry had a 97% positive predictive value for detecting moderate to severe OSA when compared to PSG. Nixon et al. [10], proposed the McGill Oximetry Score (MOS) to detect and grading severity of paediatric OSA, to prioritize treatment and to plan perioperative care. Children with inconclusive oximetry studies still required PSG to rule out OSA.

At-home pulse oximetry has an excellent night-to-night consistency when evaluated as a diagnostic test for OSA and when evaluated as a severity assessment tool. Individual oximetry metrics showed variable correlations but the desaturation index (DI_4_) was highly correlated across nights [13].

The MOS correlates with well-accepted PSG metrics for OSA severity. The different categories of MOS approximately correspond to an apnea index (AHI) respectively of 4.1, 12.6, 13.3, 39.9 episodes/h [10]. Children with an abnormal MOS were significantly more likely to have an AHI ≥ 5 episodes/h than children with an inconclusive MOS [14]. Previous studies have clearly stated that an AHI of ≥5.0 episodes/h is an important cut-off for treatment in children with sleep disorders of breathing (SDB) [15].

Seasonal variability on OSA has already been studied by polysomnography both in adults and children [16–20]. Winter and spring season emerged as critical periods. In children, winter season and the younger age appeared to be associated with higher AHI. Especially for children, repetitive viral infections and allergies were postulated as major causes of winter seasonal OSA worseness. Unfortunately, no data are available for at-home pulse oximetry performed in children referred for suspected OSA [16–20]. However, we believe that it is important for clinicians to know all factors that can affect the results of all diagnostic tests for a more appropriate interpretation which will determine a correct medical action. This could be useful especially in context where polysomnography is not available.

Therefore, the major aims of this study were: a) to examine the presence of inter-subjects at-home pulse oximetry metrics seasonal variability, b) to evaluate seasonal differences in the prevalence of an abnormal McGill Oximetry score and finally, c) to assess the influence of factors such as age, sex, BMI Z-score, and asthma on the at-home pulse oximetry results.

## Methods

All children (range 1–17 years old), referred for suspected OSA to our paediatric sleep laboratory in Rome, who underwent at-home nocturnal pulse oximetry from 2009 to 2015, were included.

Patients underwent at-home pulse oximetry when they were otherwise healthy, had not a clinically evident respiratory infection or illness in the previous 2 weeks and were not under antibiotics treatment.

Exclusion criteria were the presence of congenital or genetic abnormalities, significant cardiorespiratory disease, neurological or neuromuscular conditions and/or global developmental delay. Pulse - oximetry with recordings duration < than 6 h were also excluded.

The study was approved by the Bambino Gesù Children’s Hospital Scientific Board (Rome, Italy) and parents signed an informed consent.

### Pulse Oximetry protocol

Pulse oximetry was performed as described by Brouillette et al. [9] and Nixon et al. [10]. Parents and children came to the sleep laboratory. Parents were instructed on how to perform pulse oximetry testing and completed a questionnaire including demographic, clinical data and questions regarding their child’s usual sleep and breathing pattern.

We performed a complete physical examination on each child. Weight was assessed by a digital scale; height by a stadiometer. Body Mass Index (BMI) Z-score was calculated for all patients.

Parents took the oximeter at home, performed the study, and then returned the oximeter to our sleep laboratory. Motion-resistant pulse oximeters set for a 2-s averaging time for haemoglobin saturation (SpO_2_) (RAD 5, Masimo, Irvine) were used. The pulse oximeters used a fixed 7-s averaging time for pulse rate (PR) and stored in memory SpO_2_ and PR values every 2 s.

#### Pulse oximetry variables

Saturation and PR data were extracted and analyzed using Profox Oximetry Software, Version Masimo 0706.05D. For this study were considered total effective recording time (TERT), mean oxygen saturation (mean SpO_2_%), nadir SpO_2_ (%), Time spent with SpO_2_ < 90% (%TERT); number of SpO_2_ dips ≥4%/hrs of study (DI_4_), mean PR (bpm). Periods of oximetry recording were excluded from analysis if the oximeter quality signal indicated low signal IQ™ (Masimo), low perfusion, unrecognized, defective or no sensor, interference, or ambient light.

#### Pulse oximetry variables according to the McGill Oximetry score classification categories

Pulse oximetry recordings were evaluated for patterns suggestive of paediatric OSA and for OSA severity grading. Oximetry recordings were scored according to the frequency and depth of desaturation episodes and the number of desaturation clusters. A desaturation was defined as a decrease in SaO_2_ of ≥4%; a cluster of desaturations was defined as ≥5 desaturations occurring in a 10- to 30-min period [9]. Oximetry recordings that had at least three clusters of desaturation events and at least three desaturation of <90% were considered as diagnostic of OSA [10]. Recordings that did not meet these diagnostic criteria were regarded as inconclusive. This method already showed high positive predictive value (PPV, 97%) versus PSG, considered as the reference standard [10]. The McGill Oximetry Score (MOS) was used to assess severity of OSA as previously described [10]. MOS of 2, 3, and 4 were considered diagnostic for increasing severity of OSA, while a MOS of 1 was considered as inconclusive and unable to rule out OSA [10]. Briefly, MOS category 2 had at least three clusters of desaturation events and at least three dips in saturation of <90%; MOS category 3 had at least three clusters of desaturation events and at least four dips in saturation of <85%; and MOS category 4 had at least three clusters of desaturation events and at least four dips in saturation of <80% [10].

### Season’s definitions

Data from each subject were grouped into the season during which the at-home pulse oximetry was performed. Seasons were defined as follow: Spring: March Equinox to June Solstice; Summer: June Solstice to September Equinox; Fall (autumn): September Equinox to December Solstice; and, Winter: December Solstice to March Equinox.

#### Poly(somno)graphy

In our clinical setting, children >1 year of age without significant co-morbidities, referred for suspected OSA, are firstly screened by an at-home pulse oximetry. Children having an abnormal MOS receive as quickly as possible an extensive evaluation aimed to identify the causes of OSA and to establish an individualized care plan. In cases of inconclusive studies, when suggested by the sleep medicine physicians or requested by the referring otolaryngologist, children are evaluated by PSG using standard techniques (PSG) or cardiorespiratory sleep studies (PG) [1, 7]. One or more mixed obstructive apnoea or hypopnoea events per hour is considered abnormal and diagnostic of OSA in children [1, 7, 10].

## Data analysis

Statistical analysis was performed with Statistica 7.0 (StatSoft corp., Tulsa, OK, U.S.A). Continuous data were summarised as mean ± standard deviation (min – max), unless otherwise specified. Continuous variables were assessed using t tests or Mann-Whitney tests, when normally or non-normally distributed. Continuous non-parametric variables for seasonal groups were assessed using Kruskal-Wallis test. Chi-squared test was used for comparing proportions. A logistic regression model was performed to assess the relationship between patients’ main characteristics, seasons and the likelihood to have an abnormal MOS. Variables considered in the model included age, sex, BMI z-score, asthma, seasons and MOS in order to adjust the analysis for the potential confounding effect. For all analysed parameters, *p* < 0.05 was considered statistically significant.

## Results

A total of 781 children (502, 64.3% Males) were recruited. Their mean age was 4.9 ± 2.5 (1.0–16.1) years and BMI Z-score was −0.3 ± 2.8 (−3.9–2.9). Amongst overall children studied, 104 had an abnormal MOS (13.3%). Details on pulse oximetry data are shown in Table 1.

**Table 1:** Children’s main characteristics and at-home pulse oximetry results

	All patients
Number of patients	781
Male (N° of pts.; %)	433 (55.4%)
Age (years)	4.9 ± 2.5 (1.0–16.1)
BMI Z-score	−0.3 ± 2.8 (−3.9–2.9)
Obesity (N° pts.; %)	78 (10.0%)
Asthma (N° pts.; %)	168 (21.5%)
Allergy (N° pts.; %)	93 (11.9%)
Malocclusion (N° pts.; %)	70 (9.0%)
TERT (hrs)	8.0 ± 1.2 (6.0–13.1)
mean SpO_2_ (%)	97.9 ± 1.3 (86.5–99.9)
nadir SpO_2_ (%)	90.0 ± 8.7 (6.0–99.0)
SpO_2_ < 90% (% TERT)	0.4 ± 3.2 (0.0–23.9)
DI_4_ (Falls in saturation by ≥4%/hrs)	2.7 ± 7.1 (0.6–113.4)
mean PR (BPM)	87.1 ± 12.6 (51.0–141.0)

Data expressed as mean ± SD, unless otherwise specified. *TERT* Total Effective Recording Time, *SpO*_*2*_ Oxygen Saturation, *DI*_*4*_ Oxygen Desaturation ≥4% Index, *PR* Pulse Rate

### Pulse oximetry parameters according to an inconclusive or abnormal MOS

The main parameters from nocturnal at-home pulse oximetry divided by categories of MOS are summarized in Table 2. Patients with abnormal MOS were significantly younger (*p* < 0.0001) and had a slightly higher TERT (*p* < 0.02) than those with inconclusive MOS. As expected, in children with abnormal MOS, mean and nadir SpO_2_ were significantly lower and time spent with SpO_2_ < 90% (% TERT) significantly higher (*p* < 0.0001 for all parameters). For children with an abnormal MOS, similarly, the number of episode of desaturations ≥4% (DI_4_) and the mean Pulse Rate were significantly higher (*p* < 0.0001 for both parameters).

**Table 2:** At-home pulse oximetry in children with inconclusive and abnormal MOS

	McGill Oximetry Score (MOS)	
	Inconclusive	Abnormal
Number of patients	677	104
Male (N° of pts.; %)	433 (55.4%)	69 (66.3%)
Age (years)^*^	5.1 ± 2.5 (1.0–16.1)	3.6 ± 2.0 (1.2–15.3)
BMZ Z-score	−0.3 ± 2.8 (−3.7–2.9)	−0.2 ± 2.7 (−3.9–2.8)
TERT (hrs)^**^	8.0 ± 1.2 (6.0–13.1)	8.3 ± 1.3 (6.1–12.2)
mean SpO_2_ (%)^*^	98.1 ± 1.3 (94.4–99.9)	96.5 ± 2.2 (86.5–99.3)
nadir SpO_2_ (%)^*^	92.4 ± 3.9 (54.0–99.0)	74.9 ± 14.4 (6.0–93.0)
SpO_2_ < 90% (% TERT)^*^	0.0 ± 0.1 (0.0–2.1)	3.2 ± 8.4 (0.0–73.9)
DI_4_ (Falls in saturation by ≥4%/hrs)^*^	1.0 ± 1.6 (0.0–15.9)	13.7 ± 15.0 (2.1–113.4)
mean PR (BPM) *	85.5 ± 11.9 (51.0–141.0)	97.4 ± 12.4 (62.7–127.0)

Data expressed as mean ± SD, unless otherwise specified. *TERT* Total Effective Recording Time, *SpO*_*2*_ Oxygen Saturation, *DI*_*4*_ Oxygen Desaturation ≥4% Index, *PR* Pulse Rate, *P* values ^*^ < 0.0001, P values ^**^ < 0.02

### Pulse oximetry metrics according to season of recordings

The main parameters from nocturnal at-home pulse oximetry divided by season of recordings are summarized in Table 3. Patients with pulse-oximetry recordings performed during winter were significantly younger (*p* < 0.02). During summer, recordings had a slightly shorter TERT than in the other seasons (*p* < 0.05). Exams performed during winter and spring showed a lower mean SpO_2_ (%) than during autumn (*p* < 0.002). During winter the nadir SpO_2_ (%) was significantly lower (*p* < 0.005) and DI_4_ (Falls in saturation by ≥4%/h) significantly higher than during all others seasons (*p* < 0.005). Time spent with SpO_2_ < 90% (%TERT) was significantly lower during winter compared to autumn and spring (*p* < 0.02). Mean pulse rate was significantly higher during winter compared to other seasons (*p* < 0.001).

**Table 3:** Patient’ main characteristics and pulse oximetry data by seasons

	Seasons				
	Autumn	Winter	Spring	Summer	P values
Number of patients	198	145	266	172	–
Male (N° of pts.; %)	128 (64.4%)	86 (59.3%)	173 (65%)	115 (66.9%)	NS
Age	5.1 ± 2.8 (1.0–16.11)	4.3 ± 1.9 (1.0–13.2)	5.1 ± 2.4 (1.2–15.3)	5.0 ± 2.6 (1.0–14.6)	W vs A, Spr, Su < 0.02
BMI Z-score	−0.3 ± 2.7 (−3.9–2.7)	−0.2 ± 2.7 (−3.5–2.8)	−0.3 ± 2.9 (−3.2–2.9)	−0.3 ± 2.8 (−3.4–2.7)	NS
TERT (hrs)	8.0 ± 1.2 (6.0–13.1)	8.1 ± 1.2 (6.0–12.2)	8.1 ± 1.2 (6.0–11.4)	7.8 ± 1.1 (6.0–11.1)	Su vs W, Spr < 0.05
mean SpO_2_ (%)	98.1 ± 1.3 (86.5–99.9)	97.7 ± 1.5 (92.0–99.8)	97.8 ± 1.0 (88.3–99.9)	98.0 ± 1.0 (92.2–99.9)	A vs W, Spr < 0.002
nadir SpO_2_ (%)	90.8 ± 7.7 (29.0–99.0)	87.9 ± 11.1 (6.0–97.0)	90.2 ± 6.2 (6.0–9 8.0)	90.8 ± 5.8 (64.0–98.0)	W vs A, Spr, Su < 0.005
SpO_2_ < 90% (% TERT)	0.6 ± 5.4 (0.0–73.9)	0.8 ± 3.2 (0.0–25.3)	0.4 ± 0.6 (0.0–25.5)	0.1 ± 0.4 (0.0–3.7)	W vs A, Spr < 0.02
DI_4_ (Falls in saturation by ≥4%/hrs)	2.2 ± 5.0 (0.0–42.1)	4.5 ± 10.9 (0.0–113.4)	2.6 ± 4.2 (0.0–91.3)	2.0 ± 2.8 (0.0–33.6)	W vs A, Spr, Su < 0.005
mean PR (BPM)	87.0 ± 13.2 (53.3–141.0)	91.2 ± 13.1 (60.0–138.0)	86.0 ± 11.9 (54.0–122.0)	85.5 ± 12.8 (51.0–114)	W vs A, Spr, Su < 0.001
Diagnostic Studies (N°,%)	20 (10.1%)	32 (22.1%)	33 (12.4%)	19 (11.0%)	Chi-squared = 12.36; p < 0.01

Data expressed as mean ± SD, unless otherwise specified. *TERT* Total Effective Recording Time, *SpO*_*2*_ Oxygen Saturation, *DI*_*4*_ Oxygen Desaturation ≥4% Index, *PR* Pulse Rate, *A* Autumn, *W* Winter, *Spr* Spring, *Su* Summer

### McGill oximetry score according to seasons

As shown in Table 3, an abnormal MOS was detected significantly more often during winter than all other seasons (*p* < 0.01). Moreover, patients performing pulse oximetry during winter were nearly 2 times more likely to have an abnormal MOS [aOR 1.949 (95% CI, 1.2074–3.146] than in the other seasons (Table 4).

**Table 4:** Summary of factors potentially influencing McGill Oximetry Score

Variables	aOR	95% CI		P value
		Lower	Upper	
Age (yrs)	0.688	0.601	0.789	<0.0001
Winter	1.949	1.207	3.146	<0.01
Asthma	0.596	0.333	1.064	0.0799
BMI Z-score	1.024	0.937	1.120	0.6005
Sex (Female)	0.945	0.599	1.4902	0.8079

*aOR* adjusted Odds Ratio

The logistic regression (Table 4) where dependent variable encoding was inconclusive versus abnormal MOS, showed that younger age and winter season were associated with a higher risk to find an abnormal at-home pulse oximetry. None of the other variables included (sex, BMI Z-score, asthma) was associated with a higher risk to find an abnormal pulse oximetry.

#### Poly(somno)graphy

Forty-six children performed a P(S)G. Of them, 34 (73.9%) were males and their mean age was 4.9 ± 2.9 (1.0–12.5) years. Their mean mixed-obstructive apnea/hypopnea index (MOAHI) was 7.1 ± 10.9 (0.0–53.8) events/h. Fourteen children were studied during autumn, 3 children during winter, 16 children during spring, and 13 children during summer. Seven children showed a normal P(S)G (MOAHI <1 events/h); 22 children showed mild OSA (MOAHI 1 to 4.9 events/h); 8 children showed moderate OSA (MOAHI 5.0 to 9.9 events/h); 9 children showed severe OSA (MOAHI ≥10 events/hrs). Comparing children with mild to moderate OSA with children with severe OSA, we didn’t find statistically significant differences for: age [respectively, 4.6 ± 2.6 (1.0–11.3) vs 5.0 ± 2.8 (1.9–10.7) years; *p* = 0.7117); presence of asthma (respectively, 1/8 vs 9/21, Chi squared = 1.296; *p* = 0.2559); allergies (respectively, 0/9 vs 4/26, Chi squared = 1.337; *p* = 0.3332), and malocclusions (Chi squared = 3.479; *p* = 0.6217).

## Discussion

In this study on a large cohort of children, we investigated factors influencing the ability of at-home pulse oximetry to identify children with moderate to severe OSA. Winter season was associated with an increased prevalence and risk of having an abnormal at-home pulse oximetry. Younger age was also found as a factor influencing the abnormality of pulse oximetry.

Previous studies, performed both in children and adults, assessed that seasonality play a role as a factor favoring obstructive sleep apnea. These studies were based on objective assessment using polysomnography [16–20].

Gozal et al. [17], studied 1051 children with a mean age of 7 years. Authors found a significant higher mean AHI and a significant lower nadir SpO_2_ in winter and spring compared to summer and autumn.

Nakayama et al. [18], studied 554 children with a mean age of 6.6 years. In children < than 6 years old, they found the highest AHI in March and the lowest AHI in August. Differently, in the group ≥6 years old, the highest AHI was found in January, while the lowest AHI emerged in December.

Walter L et al. [19], studied 257 children aged from 3 to 12-year-old. They reported the highest AHI in winter and spring compared to summer and autumn.

Greenfeld M et al. [20], studied 2178 children and adolescents with a mean age of 4.9 years. They found a significantly higher mean AHI in winter compared to summer, particularly in children younger than 5 years.

Cassol CM et al. [16], retrospectively analyzed 7.523 adult patients. They reported a circannual pattern of AHI, with acrophase in winter and nadir during summer. The seasonality was still significant even after adjusting for sex, age, BMI, neck circumference, and relative air humidity. The AHI correlated inversely with ambient temperature and directly with atmospheric pressure, relative air humidity, and carbon monoxide levels.

With this background, we wondered whether similar findings on seasonality and other risk factors would be found when using pulse oximetry and the McGill oximetry score to diagnose OSA. We therefore examined a large consecutive case series of children evaluated for suspected OSA.

Evaluating pulse oximetry metric results by season of recordings, we found that winter was characterized by a lower nadir SpO_2_, a greater amount of time spent with SpO_2_ < 90% (%TERT), and a higher DI_4_ compared to other seasons.

In our large series, the overall prevalence of abnormal at-home diagnostic pulse oximetry was 13.3%, with a peak during winter (22.1%). During winter children were nearly 2 times more likely to have an abnormal MOS compared with any other season.

Although referred to the pulse oximetry, these results are similar to those described in previous studies performed with polysomnography, and reinforce the hypothesis of a relevant seasonal influence on study performed in children evaluated for suspected OSA.

In the multiple logistic regressions, where the at-home pulse oximetry outcome was the depending variable and age, sex, BMI z-score, asthma and seasons were the independent variables included in the analysis, younger age and winter season have been associated with an increasing risk of having an abnormal at-home pulse oximetry recording (respectively *p* < 0.0001 and *p* < 0.01).

Children with an abnormal pulse oximetry were younger than those with an inconclusive test (3.6 ± 2.0 vs 5.1 ± 2.5 years of age, p < 0.0001).

These findings are consistent with previous reports. Brouillette RT et al. [21], found in 436 children referred for suspected OSA, that children in the OSA group were 1.5 years younger than those negative for OSA (300 pts.: 4.2 ± 1.7 vs 136 pts.: 5.7 ± 1.7, *p* < 0.001). In our previous study [13] on 148 children, we found that children with an abnormal at-pulse oximetry were younger than those with an inconclusive test (3.6 ± 1.6 versus 5.2 ± 2.4 years, p < 0.001). Horwood L et al. [12], in a series of 362 children confirmed that children with an abnormal pulse oximetry were younger than those with an inconclusive study [3.7 (2.8–5.9) versus 5.1 (3.5–6.8), *p* < 0.002].

The explanation of this results poses some difficulties as we can only speculate on possible causes without any objective specific measure. Gozal et al. [17], postulated that respiratory viruses or airborne allergens could promote the increases in snoring and/or higher AHI during winter and spring. Other authors suggested that in winter there is an increased adenotonsillar hypertrophy which predisposes to OSA and increases the percentage of studies diagnostic of OSA [19]. Greenfeld M et al. [20], reported that the increased prevalence of OSA is more prominent in children younger than 5 years old independently from the presence of asthma/atopy, and that viral respiratory infections are most likely the major contributor for the seasonal variability observed in pediatric SDB.

Marcus C et al. [22], in 464 children with OSA aged from 5 to 9 years demonstrated the normalization of polysomnographic findings 7 months later in children in the early-adenotonsillectomy group than in the watchful-waiting group (79% vs 46%). Although these data demonstrate the greater effectiveness of early-adenotonsillectomy, it must be pointed out that after 7-months almost 50% of children presented normalization of polysomnography without surgery. Importantly, these data suggest that a significant proportion of children may improve their OSA moving from one season to another.

As for polysomnography, this seasonal variability should always be considered for the pulse oximetry. This variability may modify the clinical management strategy for children with suspected OSA, especially in borderline cases where a watchful waiting period could be taken into account.

There are several limitations in our study. The present was a retrospective study, although we reported results from a large cohort of children. Children between 1 and 17 years of age without complex comorbidities were included. Thus, our findings may not apply to younger infants, and to children with complex comorbidities.

## Conclusions

In our large study, the overall prevalence of abnormal at-home pulse oximetry was 13.3% with the majority of OSA diagnosed during winter (22.1%). Importantly, our findings suggest that younger age is an important factor positively influencing the prevalence of abnormal at-home pulse oximetry in children. Moreover, the coldest season confirms to be a critical period for night studies therefore, seasonal differences of at-home pulse oximetry results should always be taken into account for clinical management of children with suspected OSA, especially in borderline cases.

## Abbreviations

AHI: apnea index
BMI: Body Mass Index
DI4: number of SpO2 falls ≥4%/hrs of study
MOS: McGill Oximetry Score
OSA: Obstructive Sleep Apnea
PR: Pulse Rate
PSG: Polysomnography
SDB: sleep disorders of breathing
SpO_2_: oxygen saturation
TERT: Total Effective Recording Time
